# *in vitro* Anti-Proliferative Effect of Adiponectin on Human Endometriotic Stromal Cells through AdipoR1 and AdipoR2 Gene Receptor Expression

**DOI:** 10.7508/ibj.2016.01.002

**Published:** 2016-01

**Authors:** Somayeh Bohlouli, Arezou Rabzia, Ehsan Sadeghi, Farzaneh Chobsaz, Mozafar Khazaei

**Affiliations:** 1 Dept. of Veterinary, College of Agriculture, Kermanshah Branch, Islamic Azad University, Kermanshah, Iran;; 2 Fertility and Infertility Research Center, Kermanshah University of Medical Sciences, Kermanshah, Iran;; 3 Research Center for Environmental Determinants of Health (RCEDH), Kermanshah University of Medical Sciences, Kermanshah, Iran

**Keywords:** Adiponectin, Adiponectin receptors, Endometriosis, Stromal cells

## Abstract

**Background:**

Endometriosis is a complex disorder in reproductive age women which consist of stromal and epithelial cells implantation outside the uterine cavity. Adiponectin is a member of cytokine family with various metabolic roles and proliferation inhibition of many cancer cells. The aim of the present research was to determine adiponectin effect on human endometriotic stromal cells (ESCs) proliferation and their expression of adiponectin receptors.

**Methods:**

In this experimental study, endometrial biopsies (n=7) were taken. ESCs isolation was done by enzymatic digestion and cell filtrations. ESCs of each biopsy were divided into four groups: 0 (control), 10, 100, and 200 ng/ml adiponectin concentrations in three different times (24, 48, or 72 h). The effect of adiponectin on ESC viability and expression of mRNA Adipo receptor1 (R1) and Adipo receptor2 (R2) was determined by Trypan blue staining and semi-quantitative RT-PCR, respectively. Data were analyzed by one-way ANOVA and unpaired student’s *t*-test, and *P*<0.05 was considered statistically significant.

**Results:**

Adiponectin inhibited human endometriotic stromal cell proliferation in time- and dose-dependent manners significantly (*P*=0.001). Expression of AdipoR1 and AdipoR2 gene receptors was increased in human ESCs significantly (*P*<0.05).

**Conclusions:**

Adiponectin can suppress endometriosis by inhibiting ESC proliferation and increased AdipoR1 and AdipoR2 expression.

## INTRODUCTION

Endometriosis is a chronic, benign, estrogen-dependent disorder in women at reproductive age. This disorder is defined by existing endometrial stromal and epithelial cells outside the uterine cavity, and it is accompanied by chronic pelvic pain and infertility^[^^1^^,^^2^^]^. Endometriosis is diagnosed by laparoscopy and many factors such as hormones, immune cells, cytokines, and environmental factors affect the growth of endometriosis lesion^[^^3^^,^^4^^]^.

Adipokines are members of cytokine family that are secreted by adipose cells and found in blood plasma. Adiponectin regulates important metabolic processes such as glucose level, lipid metabolism, and reproductive system functions. It has two receptors named R1 and R2^[^^5^^,^^6^^]^, and its serum level is 3-30 µg/ml in human and 3-6 µg/ml in rodents^[^^7^^]^. Adiponectin plays a pivotal role in cancers and diseases of female genital system. Decreased adiponectin level has been shown to relate with the increased risk of some diseases, including orofacial cleft^[^^8^^]^ as well as endometrial^[^^9^^]^, breast^[^^10^^]^, and prostate^[^^11^^]^ cancers. Studies have indicated that adiponectin inhibits endothelial cell proliferation^[^^12^^]^, and it is decreased in plasma serum and peritoneal fluid of endometriosis patients^[^^13^^]^. 

Endometriosis treatments include surgical resection and the consumption of estrogen inhibitors, such as aromatase inhibitors, danazol, oral contraceptive pill, progestins, GnRH agonists, progesterone antagonists, and selective estrogen modulator receptors^[^^2^^,^^4^^]^. Also, experimental studies have suggested the effectiveness of statins in suppression of endometriosis^[^^14^^]^. In histo-pathological studies of endometriosis tissue, stromal cell changes are more prominent than epithelial cells and glands. Therefore, it seems that stromal cells play a central role in endometriosis. 

The proliferation of stromal cells and its invasion into peritoneum have been observed as nodules or plaques^[^^15^^,^^16^^]^. Our previous work showed that adipo-nectin inhibits human endometrial stromal cell viability^[^^17^^]^. There are many biochemical, enzymatic and gene expression differences between stromal cell from normal endometrium and endometrium from endometriosis patients, which is named endometriotic cell^[^^18^^]^. The aim of the present study was to determine the effect of adiponectin on proliferation of endometriotic stromal cell (ESC) and gene expression of adiponectin receptor in an *in vitro* culture.

## MATERIALS AND METHODS

DMEM/F-12 and fetal bovine serum were purchased from Gibco Co. (Germany), type I collagenase from Sigma (Germany), cell strainer from BD Falcon (USA), and high-molecular-weight human recombinant adiponectin from R & D Systems (Minneapolis, MN, USA).

**Sample collection**

In this *in vitro* experimental study, endometrial biopsies were obtained from reproductive aged women (n=7, 24-38 years) with endometriosis stages III and IV. The endometriosis was recognized during diagnostic laparoscopy for infertility. All patients had not received hormonal treatment and intrauterine device during three months before surgery. The phases of the menstrual cycles were secretory. The work on human endometrial tissue was accepted by the Ethics Committee of Kermanshah University of Medical Sciences (Iran), and all patients signed an informed consent.

**Endometriotic stromal cell isolation**

Endometrial samples were collected in a sterile condition and washed to remove blood and mucosa. The tissues were chopped into tiny pieces and incubated in DMEM/F-12 with type I collagenase (2 mg/ml) at 37ºC for 60-90 min. After filtration by 70- and 40-μm cell strainers, ESCs were collected after Ficoll padding and centrifugation. ESCs were cultured in DMEM/F-12 containing 5% fetal bovine serum, 100 U/ml penicillin, and 0.1 mg/ml streptomycin. The ESCs were incubated at 37ºC in a humidified atmosphere of 95% air and 5% Co_2_. The cells reached confluence in 5-7 days and then used for the experiments. The purity of the stromal cell preparations was more than 85%, as judged by positive cellular staining for anti-vimentin antibody^[^^19^^]^.

**Cell treatment**

ESCs were plated (1.5×10^5^/well) in a 24-well culture dish. After 24 hours, the culture in serum-free media was treated with DMEM/F-12 containing one of the adiponectin concentrations (0, 10, 100, and 200 ng/ml) for three different times (24, 48, or 72 h)^[^^17^^]^. Then cell viability was determined by Trypan blue staining.

**Adiponectin receptor expression**

Total RNA was extracted from stromal cells in control and adiponectin (100 ng/ml for 48 h) groups with RNeasy plus Mini Kit (Qiagen, Germany). RT-PCR was performed using One-Step RT-PCR Kit (Qiagen, Germany). The reverse transcription step was performed at 50ºC for 30 min at the beginning of RT-PCR program. The amplification reactions were carried out with the following cycles: 95ºC for 15 min (1 cycle), followed by 30 cycles of denaturation at 94ºC for 45 seconds, annealing at 58ºC (GAPDH) and 62ºC )AdipoR1 and AdipoR2 [Adipo receptor 1 and 2]) for 90 s, template extension at 72°C for 60 s and final extension at 72°C for 10 min and incubation at 4°C for 10 min. Since less than 30 cycles did not produce PCR products of sufficient intensity, we supposed that the reactions were still in the exponential phase. Experiments were performed in triplicate to ensure reproducibility. PCR was performed using AdipoR1 (228 bp), AdipoR2 (300 bp), and GAPDH (224 bp) oligonucleotide primers. The primers used were as follows:

GAPDH forward primer: 

5'CCAGGTGGTCTCCTCTGACTTCAAC-3'

GAPDH reverse primer:

5'-AGGGTCTCTCTCTTCCTCTTGTGTGCTC-3'

AdipoR1 forward primer:

5'-AAACTGGCAACATCTGGACC-3'

AdipoR1 reverse primer:

5'-GCTGTGGGGAGCAGTAGAAG-3'

AdipoR2 forward primer:

5'- ACAGGCAACATTTGGACACA-3'

AdipoR2 reverse primer:

 5'- CCAAGGAACAAAACTTCCCA-3'

After ampliﬁcation, the PCR products were separated on 1.5 % (w/v) agarose gel and stained with 1 µg/ml ethidium bromide (Sigma, USA) and photographed under UV light using an ultraviolet trans-illuminator (UVIdoc; Uvitec, Cambridge, UK). Gel images were analyzed using the UN-SCAN-IT program. The GAPDH gene was used as a reference. Relative RT-PCR values were presented as the ratio of the AdipoR1 and AdipoR2 gene signals divided by the GAPDH signal. RT-PCRs were performed as three individual replicates^[^^17^^]^. 

**Statistical analysis**

Data were reported as means ± SEM, and statistical analysis was carried out by one-way analysis of variance (ANOVA), followed by Tukey’s test. The significance differences between two RT-PCR groups were determined using the unpaired student’s *t*-test, and *P*<0.05 was considered statisticaaly significant.

## RESULTS

**Endometriotic stromal cell** **proliferation**

**Fig. 1:**
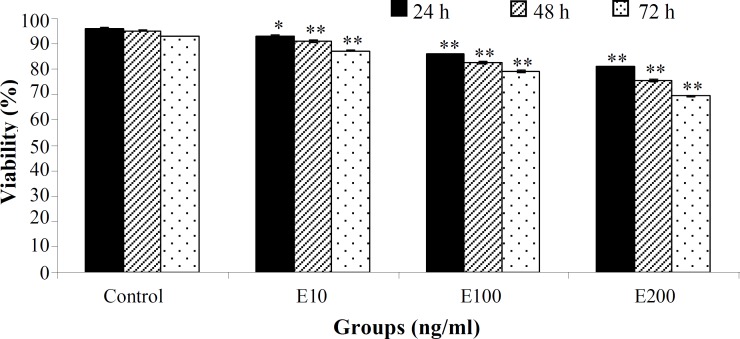
Adiponectin effect on human endometriotic stromal cells in 24, 48, and 72 hours. Adiponectin reduced the cell viability in a dose- and time-dependent manner. ^*^ and ^**^ show significantly different from the control group, respectively (*P*<0.05 and *P*=0.001).

**Fig. 2:**
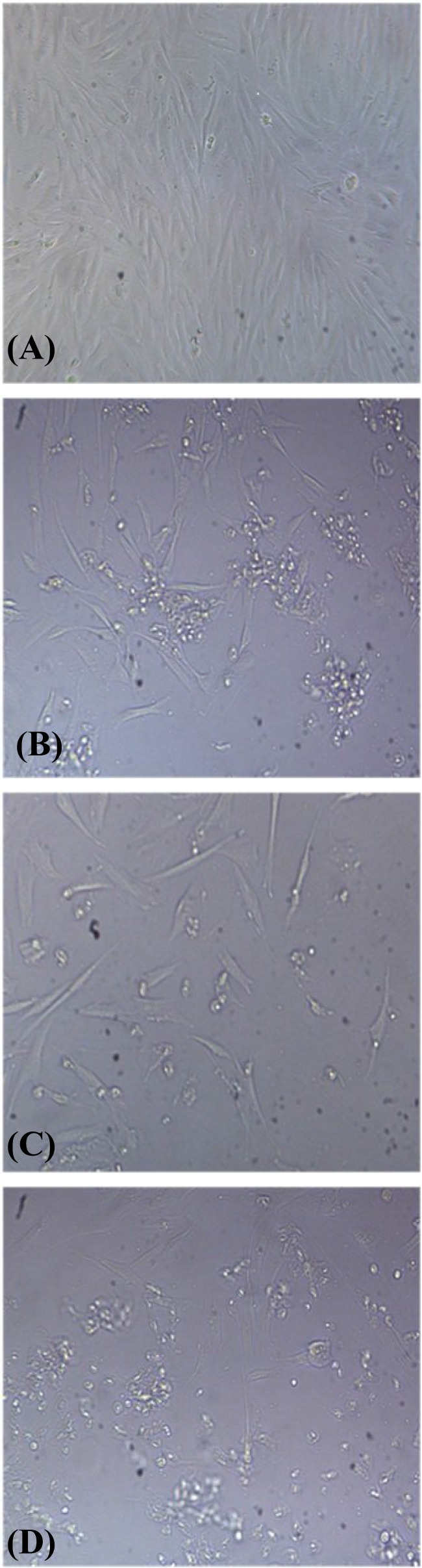
The morphology of endometriotic stromal cells in the control group. The 200 ng/ml group (A) in 24 (B), 48 (C), and 72 hours (D) (×100).

ESCs were treated with various concentrations of adiponectin (0, 10, 100, and 200 ng/ml) for 24, 48, and 72 h. In comparison to control group, ESC proliferation was reduced significantly in all concentrations in a dose and time-dependent manner (*P*=0.001) (Fig. 1). Findings of morphological consideration of ESCs showed that by increasing adiponectin dose and time duration, the cells lost their normal morphology (fibroblastic polygonal) and changed to spindle and spherical shapes. Also, dead, tiny and floating cells were increased compared to the control group, especially in 200 ng/ml concentration and 72 hours incubation (Fig. 2).

**mRNA expression of AdipoR1/R2**

**Fig. 3:**
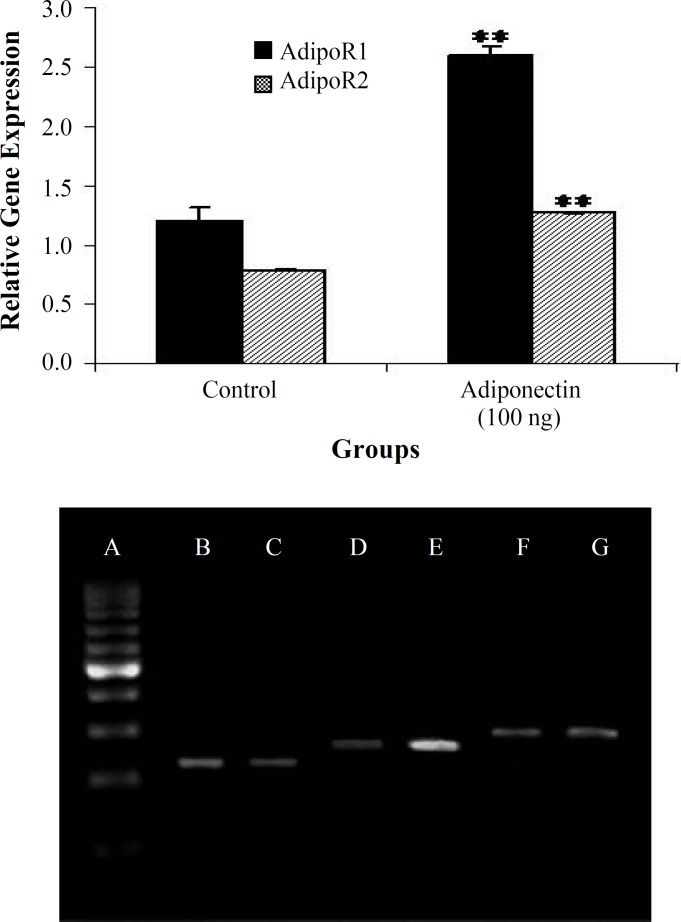
Semi-quantitative RT-PCR expression of endo-metriotic stromal cells (AdipoR1 and AdipoR2) in the control and treated groups (100 ng/ml adiponectin). A (marker 100 bp), B (GAPDH of control), C (GAPDH of treated with 100 ng/ml adiponectin), D (AdipoR1 of control), E (AdipoR1 of treated with 100 ng/ml adiponectin), F) AdipoR2 of control (, and G (Adipo R2 of treated with 100 ng/ml adiponectin). ** shows significant difference between control and 100 ng/ml adiponectin (*P*<0.001)

AdipoR1 and AdipoR2 expressions of human ESCs were performed by semi-quantitative RT-PCR. The results showed that the expression levels of mRNA of AdipoR1 and AdipoR2 in the treated group (100 ng/ml adiponectin and 48 h) was increased significantly compared to the control group (*P*<0.05) (Fig. 3).

## DISCUSSION

In this study, the effect of adiponectin on the ESC proliferation and expression of AdipoR1 and AdipoR2 receptors was investigated *in vitro*. Adiponectin caused a significant reduction in viability of ESCs in dose- and time-dependent manners. Reduction of serum adiponectin concentrations has been observed in endometriosis women, especially in stages III and IV^[^^12^^]^. In addition, in advanced stages of endometriosis, a significant reduction of adiponectin was found in peritoneal fluid of endometriosis patients^[^^20^^]^. Evidence has indicated that adiponectin plasma concentrations are decreased in genital cancers, such as estrogen-related cancer as well as endometrial^[^^9^^,^^21^^]^, breast^[^^10^^]^, and prostate cancers^[^^11^^]^.

In a study similar to the present work, it has been reported the suppressive effect of adiponectin on proliferation and the growth of endometrial carcinoma cell lines (HEC-1-A and RL95-2) and its apoptosis induction in these two categories of endometrial cancer^[^^22^^]^. In studies on the breast cancer (MCF7 cell line), adiponectin growth suppression and the creation of apoptosis^[^^23^^]^ were reported, as well as the *in vitro* growth inhibition of breast cancer cells lines: T47D and MDA-MB-231 cells by adiponectin was shown^[^^24^^]^. Also, another study indicated the growth-inhibitory effects and anti-proliferative properties of adiponectin and the reduction of the number of viable cells in human trophoblastic cell lines (JEG-3 and BeWo) and primary trophoblast cells^[^^25^^]^. Decreasing the pro-liferation of colon cancer cells lines HCT16 and HT29 by adiponectin in a cell culture was also documented^[^^26^^]^.

Due to the dependency of endometriosis on estrogen hormone and its similarity to estrogen-dependent cancers, such as endometrial and breast cancers, the results obtained in the current study confirmed the above-mentioned studies. This confirmation was based on this fact that adiponectin reduces the proliferation of different cell types of reproductive system cancer and affirms the functional role of adiponectin in the inhibition of cell proliferation.

Adiponectin applied its biological effects through its receptors (AdipoR1 and AdipoR2). AdipoR1and AdipoR2 mRNA expression of genes were investigated in a concentration of 100 ng/ml adiponectin in ESCs. Based on our findings, adiponectin significantly affects the genes expression of these receptors (AdipoR1and AdipoR2) in ESCs and causes a surge in the gene expression of both adiponectin receptors in endo-metriosis sample. Increasing receptor expression in endometriotic samples reached to the level of the normal rate. Reduction in the amount of serum adiponectin in these patients, and their sensitivity to the presence of adiponectin can be due to increased gene expression of adiponectin receptors in endometriotic samples. Observing the expression of adiponectin receptors in these cells confirms the results of a previous study in which the presence of these two receptors in the endometrial epithelial and stromal cells in both proliferative and secretory phases was indicated^[^^27^^]^.

Some investigations have demonstrated that Adiponectin decreases the cell proliferation in human endometrial cancer tissue through adiponectin receptors, and the expression rate of AdipoR1 receptor is more than AdipoR2, but in normal and non-cancerous tissues, they did not have a significant difference^[^^22^^,^^28^^]^. Also, it has been reported that adipo-nectin increased the expression level of AdipoR1 and AdipoR2 receptors in primary trophoblast cells and the cell line, BeWo, but this increase was not statistically significant^[^^25^^]^.

Regarding the pivotal role of stromal cells in endometriosis and decreasing the amount of serum adiponectin in endometriosis, is a suitable marker for endometriosis and it can consider as a therapeutic option for endometriosis treatment. The study of adiponectin effect on endometriotic tissue in other experimental and animal models of endometriosis is suggested.
